# Parental Technoference and Child Problematic Media Use: Meta-Analysis

**DOI:** 10.2196/57636

**Published:** 2025-01-22

**Authors:** Jinghui Zhang, Qing Zhang, Bowen Xiao, Yuxuan Cao, Yu Chen, Yan Li

**Affiliations:** 1 Shanghai Institute of Early Childhood Education Shanghai Normal University Shanghai China; 2 Psychology Department Carleton University Ottawa, ON Canada

**Keywords:** parental technoference, child, problematic media use, meta-analysis, children, youth, adolescents, phubbing, screen distraction, systematic reviews, interventions, mental health, digital health

## Abstract

**Background:**

Parental technoference, the interruption of parent-child interactions by technology, has been associated with negative outcomes in children’s media use. However, the magnitude of this relationship and its moderating factors remain unclear.

**Objective:**

This study aims to systematically examine the relationship between parental technoference and child problematic media use, as well as to identify moderating factors such as age, parental technoference group, study design, and type of problematic media use.

**Methods:**

Following PRISMA (Preferred Reporting Items for Systematic Reviews and Meta-Analyses) guidelines, a comprehensive literature search was conducted up to August 2024 across multiple databases, including Web of Science, EBSCO, ProQuest, PubMed, PsycINFO, and China National Knowledge Infrastructure, using predefined search strings. A total of 53 studies with a total of 60,555 participants (mean age of 13.84, SD 1.18 years) were included. Inclusion criteria comprised studies involving children under the age of 22 years, assessing the association between parental technoference and child problematic media use with valid measures, and reporting necessary statistical data. Exclusion criteria included studies focusing on other child outcomes, having sample sizes <30, or being case reports or review papers. The risk of bias was assessed using the Quality Assessment Tool for Observational Cohort and Cross-Sectional Studies. A random-effects meta-analysis was performed using R (version 4.2.1; R Foundation for Statistical Computing) with the *meta* and *metafor* packages to evaluate the association and conduct moderator analyses.

**Results:**

The meta-analysis identified a significant positive association between parental technoference and child problematic media use (*r*=0.296, 95% CI 0.259-0.331). Moderator analyses revealed that both parental technoference group (*P*<.001) and study design (*P*=.008) significantly influenced this relationship. Specifically, the association was stronger when both parents engaged in technoference compared to when only 1 parent did, and in cross-sectional studies compared to longitudinal studies. Age, gender, publication status, and type of problematic media use did not significantly moderate the relationship (all *P*>.05).

**Conclusions:**

This meta-analysis provides robust evidence of the association between parental technoference and child problematic media use. The findings highlight the need for family-based interventions and underscore the importance of longitudinal research to understand the temporal dynamics of this relationship better.

**Trial Registration:**

PROSPERO CRD42023471997; https://www.crd.york.ac.uk/prospero/display_record.php?RecordID=471997

## Introduction

### Background

Problematic media use in children refers to excessive screen engagement that disrupts functioning and development, transcending mere time metrics [1,2]. Globally, problematic media use is on the rise [3,4], with significant implications for the developmental health of children and adolescents [5-10]. A key factor implicated in the rise of problematic media use among youth is parental technoference. Technoference, a term coined by combining “technology” and “interference,” refers to the disruption or disturbance of daily interpersonal communication and shared time caused by digital and mobile technology devices [11,12]. Parental technoference, which refers to the interruptions in parent-child interactions due to digital and electronic media devices, is a concern that has been increasingly recognized within the domain of family dynamics [13-15]. Numerous empirical studies have explored the relationship between parental technoference and child problematic media use, yet the findings have been inconsistent, possibly due to variations in research methodologies and sample characteristics [2,16-18]. Despite the growing body of literature, there is a conspicuous absence of systematic reviews or meta-analysis studies aimed at synthesizing these findings to address the discrepancies and draw reliable conclusions. To fill this gap, this study used a meta-analysis method to quantitatively integrate the extant research on this topic. Our objectives are twofold: to establish a more robust and reliable estimate of the effect size of parental technoference on child problematic media use, and to identify potential moderators that may influence the strength of this relationship. Through this work, we aim to contribute valuable insights into the dynamics of family media use and to promote healthier media habits that support the well-being of children and adolescents.

### Association Between Parental Technoference and Child Problematic Media Use

Parental technoference may affect child problematic media use, and this association can be explained through various theories and psychological processes. First, the interactional theory of childhood problematic media use [19] posits that problematic media use in children is influenced by both individual and contextual factors. It suggests that children’s media use patterns are shaped by their interactions with their environment, including parental behaviors. Specifically, parental technoference may lead to unclear media use rules in the family environment and reduce interaction and communication between parents and children [11,20-22]. In such cases, children may be more inclined to engage in excessive media use to compensate for the reduced parent-child interaction caused by parental technoference. Indeed, research has found that poor parent-child relationships are a significant risk factor for children’s problematic media use [23,24]. Second, based on the accept-rejection theory [25], children’s behavior is influenced by their perception of acceptance or rejection from significant others, especially their parents. When parents are preoccupied with technology and fail to provide emotional availability and responsiveness, children may perceive this as rejection or neglect. In response, children may seek alternative sources of comfort and stimulation, such as excessive media use, to compensate for the lack of emotional connection with their parents. Thus, parental technoference can contribute to problematic media use as a coping mechanism for perceived parental rejection. Finally, social learning theory [26] emphasizes the role of observational learning and modeling in shaping behavior. According to this theory, children learn by observing and imitating the behaviors of others, particularly their parents. When parents engage in excessive media use or prioritize technology over other activities, children are more likely to adopt similar behaviors. Parental technoference, by modeling problematic media use, may contribute to the development of problematic media use in children through observational learning processes. In conclusion, parental technoference can influence child problematic media use through multiple theoretical perspectives. It disrupts parent-child interactions, may contribute to children’s perception of rejection, and models problematic media use.

Extensive empirical research has investigated the relationship between parental technoference and child problematic media use, including smartphone addiction [18,27-32], internet gaming disorder [33-37], social networking site addiction [2,38], and short-form video addiction [39]. The findings consistently indicate a significant positive correlation between parental technoference and child problematic media use, raising concerns for children across different age groups, from children with an average age of 10.33 (SD 0.98) years [37] to adolescents with an average age below 20 years [39-41]. However, there are fair-sized discrepancies in the magnitude of association between the same parental technoference and child problematic media use in the literature. For example, the correlation between parental technoference and child problematic media use ranged from 0.11 [17] to 0.54 [16]. These conflicts may be due to differences in study design, study characteristics, and result characteristics.

### Impact of Moderator Variables

Gender differences in children’s susceptibility to parental technoference and its association with problematic media use have been extensively documented in the literature. For instance, Xie et al [31] found that gender moderates the relationship between parental technoference and smartphone addiction in children, with a greater impact on smartphone addiction observed in boys compared to girls. However, Wang et al [42] did not find gender differences in the relationship between parental technoference and child problematic smartphone use. This discrepancy may be due to the uncertain nature of gender differences in problematic media use. Some studies have reported a significant correlation between gender and children’s problematic media use. Among these studies, some have indicated a more severe tendency toward problematic media use in boys compared to girls [36], while others have found the opposite [35]. Additionally, some studies have not established a significant relationship between parental technoference and child problematic media use [2,29,43]. Overall, the impact of parental technoference on child problematic media use may differ between boys and girls, although a definitive conclusion is currently lacking. Therefore, in this study, we examined the moderating effect of gender.

In addition, the age of children may also play a moderating role in the relationship between parental technoference and child problematic media use. On one hand, parental technoference may be related to the age of the child. Previous studies have found a significant positive correlation [2], a significant negative correlation [37], and a nonsignificant correlation [29,35,36] between parental technoference and child age. This variation may stem from the fact that each empirical study has only investigated a limited age range, failing to examine the relationship between parental technoference and child age from a broader perspective. On the other hand, the age of children may also influence their problematic media use. For example, researchers have found a significant positive correlation between child age and problematic media use [28,31,44], indicating that as children get older, their problematic media use becomes more severe. In summary, both parental technoference and child problematic media use vary with the child’s age. Therefore, in this study, we also examined whether child age moderates the association between parental technoference and child problematic media use.

Moreover, this study aims to explore the separate effects of parental technoference on problematic media use in children. It is well documented that fathers and mothers often have different parenting roles and practices, which can influence children’s development in unique ways [45,46]. For example, McDaniel and Radesky [15] observed a positive association between mother technoference in parenting and children’s externalizing and internalizing behaviors, while father technoference in parenting did not show a significant correlation with the child’s problem behavior. Thus, it is imperative to separately examine the influences of father and mother technoferences on children’s development. Notably, existing research has predominantly focused on the relationship between technoference from both parents and child problematic media use [2,18,28,29,31,42,44], without distinguishing the specific impacts of fathers and mothers. Therefore, this study aims to fill this gap by exploring the moderating effects of parental technoference groups on child problematic media use.

Finally, we examine the impact of different types of child problematic media use, publication status, and study design on the relationship between parental technoference and child problematic media use. Specifically, we investigated whether various types of problematic media use influence this relationship. Furthermore, we compared the findings of published studies with those of unpublished master’s thesis to determine the consistency of conclusions. Additionally, we examined potential differences in conclusions between cross-sectional and longitudinal research in the context of parental technoference studies.

### Objectives

As discussed earlier, the empirical research investigating the relationship between parental technoference and child problematic media use has yielded inconsistent results. To our knowledge, there are no meta-analysis studies in this area to explain these differences. Therefore, this study performed a meta-analysis to synthesize the findings of previous studies examining the association between parental technoference and child problematic media use to better understand the associations between these constructs. The meta-analysis aims to address two core research questions.

- What is the overall relationship between parental technoference and child problematic media use?
- Are there any moderating variables that influence the relationship between parental technoference and child problematic media use?

We also explored potential moderating variables, such as the child’s age, gender, type of problematic media use, parental technoference group, publication status, and research design.

## Methods

### Study Design

The meta-analysis followed the Preferred Reporting Items for Systematic Review and Meta-Analysis (PRISMA) guidelines [47]. The PRISMA–Individual Participant Data checklist for this review is presented in Multimedia Appendix 1. To ensure transparency and avoid unintentional duplication of effort, the protocol for this meta-analysis was registered in the International Prospective Register for Systematic Reviews (PROSPERO CRD42023471997).

### Search Strategy

A systematic literature search was conducted based on the PRISMA statement [47]. Multiple electronic databases, including Web of Science, EBSCO, ProQuest, PubMed, PsycINFO, and China National Knowledge Infrastructure, were searched using a predefined search string. The search terms consisted of three elements: (1) parent (eg, parent* OR parental* OR caregiver* OR guardian* OR dad* OR father* OR mom* OR mother* OR family); (2) child (eg, child* OR infant* OR baby OR babies OR toddler* OR preschool* OR kid* OR youth* OR teen* OR adolescent* OR young*); and (3) technoference (eg, technoference* OR phubbing* OR “technology interference*” OR “distraction with phone*” OR “digital distraction*” OR “smartphone distraction*” OR “device distraction*” OR “technology interruption*” OR “digital interruption*” OR “smartphone interruption*” OR “device interruption*” OR “parental media use*” OR “parental smartphone use*” OR “parental device use*”). The search strategy for all databases is presented in Multimedia Appendix 2. To avoid potential bias, outcome terms related to child problematic media use were not included in the search strategy, as suggested by Frandsen et al [48]. The last search was conducted in August 2024. Additionally, to ensure comprehensive coverage, the reference lists of eligible study reports and relevant reviews in the field of parental technoference were manually searched. This iterative process continued until no further studies could be identified.

### Inclusion and Exclusion Criteria

The inclusion criteria for the literature in this meta-analysis were as follows: (1) studies involving children under the age of 22 years; (2) investigations examining the association between parental technoference and child problematic media use; (3) use of a valid and reliable measure to assess parental technoference and child problematic media use; (4) clear reporting of correlation coefficients (*r*) between parental technoference and child problematic media use, or provision of 2-tailed *t* test values, *F* test values, or chi-square test values that could be converted into *r*; (5) reporting of the sample size; (6) inclusion of cross-sectional or longitudinal study designs; and (7) availability of studies written in either Chinese or English. Conversely, studies were excluded if they (1) assessed child outcome indicators other than problematic media use; (2) had a sample size of <30; or (3) were case reports or review papers.

### Selection Procedure

The study selection process was conducted in multiple stages following standard systematic review procedures. First, duplicate studies were removed. Subsequently, four authors (JZ, QZ, Y Cao, and Y Chen) working in pairs independently screened the remaining articles. Each record was assessed independently by 2 authors in a 2-stage process: initial screening of titles and abstracts, followed by full-text evaluation of potentially eligible studies. The screening process was conducted under blinded conditions to minimize selection bias. Any discrepancies between the raters were resolved through discussion with the corresponding author until a consensus was reached.

### Data Extraction

The following data were extracted: (1) first author names and publication year, (2) correlation coefficient, (3) the number of study samples, (4) gender distribution of children (measured by “boy ratio”), (5) average age of children, (6) measurement instrument, (7) parental technoference group (father technoference vs mother technoference vs parental technoference), (8) participant’s country, and (9) publication type (journal paper vs thesis).

In the process of data extraction, we followed specific guidelines. First, effect sizes were generated based on independent samples, with each independent sample contributing 1 effect size for a specific parental technoference and child problematic media use. Second, when 2 effect sizes from the same sample could be classified into 2 different subgroups for moderator analysis, they were considered separate. For example, if a sample reported correlation coefficients for both father and mother technoferences with children’s problematic media use separately, these were treated as independent when assessing the moderating effect of the parental technoference category. In other scenarios, to maintain the independence of effect sizes, when multiple effect sizes for the same variable within a group were reported, we used the average correlation to address dependency issues. Third, for longitudinal studies, only 1 longitudinal correlation between the initial measurement of parental technoference and the final measurement of child problematic media use was retained. Lastly, if participant characteristics, such as gender, were reported separately, they were coded as such.

To ensure coding reliability, 2 authors independently conducted the coding, and agreement levels were assessed. Interrater reliability was calculated using the intraclass correlation coefficient (ICC) for continuous variables and Cohen κ for categorical variables. For continuous variables, intercoder reliability was calculated for correlation coefficients (ICC=0.982), sample (ICC=0.997), gender (ICC=0.996), and age (ICC=1.000). For categorical variables, intercoder reliability was calculated for publication year (κ=1.000), publication status (κ=1.000), measurement of parental technoference (κ=1.000), parental technoference group (κ=1.000), and research design (κ=1.000). These results indicated excellent interrater reliability, reflecting a high level of agreement between the coders on the characteristics of the studies. Any discrepancies were resolved through discussion.

### Risk of Bias Assessment

The risk of bias for all included studies was assessed using the Quality Assessment Tool for Observational Cohort and Cross-Sectional Studies [49]. This tool comprises 14 items, each offering 5 response choices: yes, no, cannot be determined, not reported, and not applicable. A score of 1 was assigned for “yes,” while the other response choices did not receive any points. The total score was used to categorize the quality of the literature as good (total score>7), fair (total score between 5 and 7), or poor (total score<5). A high-quality study is generally characterized by minimal risk of bias. Two authors independently conducted the coding process, and their agreement on the total score demonstrated a high level of consistency (ICC=0.987). Any discrepancies in the coding process were resolved through consensus discussions. The specific details of the quality assessment for each study can be found in Multimedia Appendix 3 [2,16-18,27-44,50-80].

### Statistical Analyses

The meta-analysis was conducted using R (version 4.2.1-win; R Foundation for Statistical Computing) with the *meta* and *metafor* packages. Effect sizes were measured using correlation coefficients (*r*). Before the meta-analysis, all correlation coefficients were transformed into Fisher *z* scores. After the analysis, Fisher *z* values were converted back to Pearson correlation coefficients for easier interpretation. Given the anticipated heterogeneity in measurement methods for parental technoference and child problematic media use, as well as variations in participant characteristics (eg, gender, age, cultural background) across the included studies, we used a random effects model for our meta-analysis. Heterogeneity was assessed using the *Q* and *I*^2^ test statistics. A significant *Q* test or an *I*^2^ value above 75% indicated substantial heterogeneity, supporting the use of a random effects model [81].

For the moderator analysis, continuous moderators were analyzed using meta-regression, while categorical moderators were analyzed using subgroup analysis. The significance of moderators was assessed using the *Q* statistic. As recommended by Huang [82], each subgroup should include a minimum of 3 studies for the analysis of categorized moderating variables.

Publication bias refers to the tendency for significant results to be more likely to be published, while nonsignificant results may remain unpublished. To address publication bias, this study included both published journal papers and unpublished theses when selecting literature, which helped to control the influence of publication bias on the research findings. Additionally, to ensure the reliability of the meta-analysis results, funnel plot and Egger regression intercept were used to assess the presence of publication bias. If the funnel plot exhibited a symmetrical inverted funnel shape and the Egger regression intercept was nonsignificant, publication bias was considered negligible.

## Results

### Study Characteristics

**Figure 1:**
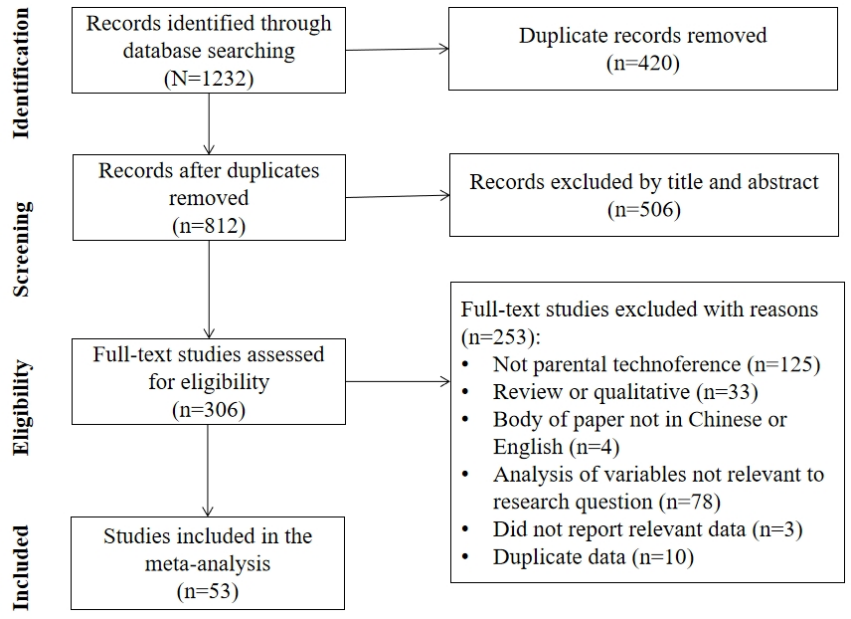
Flow diagram of study search.

Out of 1232 initially identified studies (Figure 1), 420 duplicates were excluded. After screening the titles and abstracts, 506 studies were excluded, leaving 306 studies for full-text screening. Following predefined inclusion and exclusion criteria, 253 studies were excluded. Consequently, 53 studies met all eligibility criteria and were included in the meta-analysis.

The specific information of the literature included in the meta-analysis is shown in Table 1. The meta-analysis included a total of 60 effect sizes from 53 studies, encompassing 60,555 participants. The sample sizes of the studies ranged from 227 to 4172, with an average participant age of 13.84 (SD 1.18) years. The publication years of the included studies varied from 2018 to 2024. Of the total studies, 46 were cross-sectional, while 7 were longitudinal. The literature consisted of 44 published journal papers and 9 unpublished master’s theses. The primary measurement method used in parental technoference research was questionnaire surveys. Commonly used questionnaires included the Partner Phubbing Scale, Phubbing Scale, and the Generic Scale of Being Phubbed. The analysis directly assessed parental technoference in 44 studies, while 7 studies evaluated father technoference and mother technoference separately, 1 study exclusively evaluated mother technoference, and 1 study solely evaluated father technoference. Regarding child problematic media use, the analysis covered problematic smartphone problem use, problematic internet use, problematic social media use, internet gaming disorder, and short-form video addiction, with 34 studies, 8 studies, 5 studies, 5 studies, and 1 study, respectively. Furthermore, 50 of the studies were conducted in China, while the remaining studies were from the United States, Italy, and the Netherlands. In terms of literature quality, 6 studies were rated as “fair,” while the remaining 47 were rated as “good.”

**Table 1:** Overview of studies included in the meta-analysis.

Authors (publication year)	Participants, n	Age (y), mean (SD)	Gender (boys), %	Technoference type	Questionnaire survey	Indicators	Publication type	Research design	Country	Literature quality
Chen et al (2023) [50]	728	—^a^	0.50	PT^b^	PPS^c^	PMU^d^	Journal	Cross-sectional research	China	Good
Dai et al (2024) [51]	1912	11.03 (1.13)	0.53	FT^e^ and MT^f^	PPS	PIU^g^	Journal	Cross-sectional research	China	Good
Deng and Hong (2023) [33]	855	12.25 (0.58)	0.53	FT and MT	PPS	PIU	Journal	Cross-sectional research	China	Fair
Ding et al (2018) [52]	312	12.89 (0.71)	0.55	PT	PPS	PMU	Journal	Longitudinal research	China	Good
Ding et al (2019) [53]	555	12.86 (0.71)	0.52	PT	PPS	PMU	Journal	Cross-sectional research	China	Good
Ding et al (2020) [54]	574	12.90 (0.71)	0.53	PT	PPS	PMU	Journal	Cross-sectional research	China	Good
Ding et al (2022) [55]	812	—	0.43	PT	PPS	PMU	Journal	Cross-sectional research	China	Good
Dong et al (2022) [2]	2286	13.46 (0.93)	0.49	PT	PPS	PSMU^h^	Journal	Cross-sectional research	China	Good
Fu et al (2020) [56]	2238	13.89 (2.44)	0.50	PT	PPS	PMU	Journal	Cross-sectional research	China	Good
Geng et al (2021) [27]	1447	16.15 (0.65)	0.40	FT and MT	GSBP^i^	PMU	Journal	Longitudinal research	China	Good
Geurts et al (2022) [57]	403	13.51 (2.15)	0.47	PT	SCS^j^	PSMU	Journal	Cross-sectional research	Netherlands	Good
Han (2021) [58]	582	13.53 (0.83)	0.48	PT	PPS	PIU	Thesis	Cross-sectional research	China	Good
Hernandez et al (2024) [59]	227	—	0.45	PT	SCS	PIU	Journal	Cross-sectional research	America	Good
Hong et al (2019) [44]	1721	13.69 (1.64)	—	PT	PPS	PMU	Journal	Longitudinal research	China	Good
Ji (2022) [60]	2090	16.00 (1.32)	0.44	PT	PPS	PMU	Thesis	Cross-sectional research	China	Good
Liu et al (2019) [43]	602	15.09 (2.89)	0.52	PT	PPS	PSMU	Journal	Cross-sectional research	China	Fair
Liu et al (2020) [28]	3051	13.08 (0.89)	0.49	PT	TILES^k^	PMU	Journal	Cross-sectional research	China	Good
Liu et al (2024) [61]	495	13.39 (0.77)	0.45	PT	PPS	PIU	Journal	Cross-sectional research	China	Good
Liu and Wu (2024) [62]	2465	10.43 (0.99)	0.53	PT	TILES	PMU	Journal	Cross-sectional research	China	Fair
Liu et al (2022) [63]	1202	14.54 (1.79)	-	PT	PPS	PMU	Journal	Cross-sectional research	China	Good
Liu (2021) [64]	822	13.66 (0.96)	0.50	FT and MT	PPS	PMU	Thesis	Cross-sectional research	China	Good
Ma (2023) [65]	1173	17.00 (0.91)	0.62	PT	PPS	PMU	Thesis	Longitudinal research	China	Good
Ma et al (2024) [66]	786	13.71 (1.35)	0.53	PT	PPS	PMU	Journal	Cross-sectional research	China	Good
Mi et al (2023) [16]	780	14.04 (0.93)	0.50	PT	PPS	PMU	Journal	Cross-sectional research	China	Good
Mu et al (2022) [17]	242	—	0.34	PT	PPS	SFVA^l^	Journal	Cross-sectional research	China	Fair
Niu et al (2020) [29]	726	14.55 (1.37)	0.51	PT	PPS	PMU	Journal	Cross-sectional research	China	Good
Ou and Zhu (2021) [40]	636	19.60 (1.40)	0.45	PT	PPS	PMU	Journal	Cross-sectional research	China	Fair
Pivetta et al (2024) [67]	557	15.62 (1.54)	0.69	FT and MT	PPS	IGD^m^	Journal	Longitudinal research	Italy	Good
Qiao and Liu (2020) [30]	1354	16.10 (0.96)	0.46	PT	TILES	PMU	Journal	Cross-sectional research	China	Good
Qu and Zhang (2020) [34]	318	11.56 (0.92)	0.49	FT and MT	TDIS^n^	IGD	Journal	Cross-sectional research	China	Good
Shao et al (2024) [68]	3023	10.59 (0.32)	0.57	PT	TTS^o^	PMU	Journal	Cross-sectional research	China	Good
Shen et al (2022) [35]	809	13.15 (0.62)	0.49	PT	PPS	IGD	Journal	Cross-sectional research	China	Good
Tang et al (2024) [69]	742	12.97 (0.64)	0.55	PT	PPS	PMU	Journal	Cross-sectional research	China	Good
Wang and Lei (2022) [39]	549	17.01 (2.09)	0.55	PT	PPS	PIU	Journal	Cross-sectional research	China	Good
Wang et al (2022) [38]	4172	16.41 (0.77)	0.52	FT	GSBP	PSMU	Journal	Cross-sectional research	China	Good
Wang et al (2024) [70]	234	5.02 (1.10)	0.46	PT	PPS	PMU	Journal	Cross-sectional research	China	Good
Wang et al (2023) [42]	3519	16.42 (0.77)	0.47	MT	GSBP	PSMU	Journal	Cross-sectional research	China	Good
Wang et al (2023) [71]	2260	12.76 (0.58)	0.50	PT	PPS	PMU	Journal	Longitudinal research	China	Good
Wu (2022) [72]	780	—	0.50	PT	PPS	PMU	Thesis	Cross-sectional research	China	Good
Xiao (2020) [73]	452	16.90 (0.93)	0.43	PT	PS^p^	PMU	Thesis	Cross-sectional research	China	Good
Xie et al (2019) [31]	1007	13.85 (1.53)	0.49	PT	PPS	PMU	Journal	Cross-sectional research	China	Good
Xie et al (2021) [36]	779	13.15 (0.61)	0.49	PT	PPS	IGD	Journal	Cross-sectional research	China	Good
Yang et al (2022) [74]	2220	13.97 (1.92)	0.46	PT	PPS	PMU	Journal	Cross-sectional research	China	Good
Yang et al (2022) [75]	341	13.41 (0.68)	0.52	PT	PPS	PMU	Journal	Longitudinal research	China	Good
Yang (2022) [76]	301	—	0.32	PT	PPS	PIU	Thesis	Cross-sectional research	China	Fair
Yang (2022) [77]	944	13.89 (0.87)	0.45	PT	PPS	PMU	Thesis	Cross-sectional research	China	Good
Zhang and Zhang (2020) [41]	411	19.82 (1.34)	0.38	PT	PPS	PMU	Journal	Cross-sectional research	China	Good
Zhang et al (2021) [18]	471	13.46 (1.11)	0.40	PT	PPS	PMU	Journal	Cross-sectional research	China	Good
Zhao et al (2022) [32]	931	13.54 (1.08)	—	PT	PPS	PMU	Journal	Cross-sectional research	China	Good
Zhong et al (2023) [78]	2174	13.25 (0.93)	0.51	PT	TILES	PMU	Journal	Cross-sectional research	China	Good
Zhou et al (2022) [37]	1021	10.33 (0.98)	0.56	PT	PPS	IGD	Journal	Cross-sectional research	China	Good
Zhu and Jiang (2022) [79]	1034	13.45 (0.68)	0.53	FT and MT	PPS	PIU	Journal	Cross-sectional research	China	Good
Zhu (2023) [80]	430	—	0.48	PT	PPS	PMU	Thesis	Cross-sectional research	China	Good

^a^Data missing.

^b^PT: parental technoference.

^c^PPS: Partner Phubbing Scale.

^d^PMU: problematic mobile use.

^e^FT: father technoference.

^f^MT: mother technoference.

^g^PIU: problematic internet use.

^h^PSMU: problematic social media use.

^i^GSBP: Generic Scale of Being Phubbed.

^j^SCS: Self-constructed Scale.

^k^TILES: Technology Interference in Life Examples Scale.

^l^SFVA: short-form video addiction.

^m^IGD: internet gaming disorder.

^n^TDIS: Teenagers-perceived Digital Interference Scale.

^o^TTS: The Technoference Scale.

^p^PS: Phubbing Scale.

### Analysis of Publication Bias

**Figure 2:**
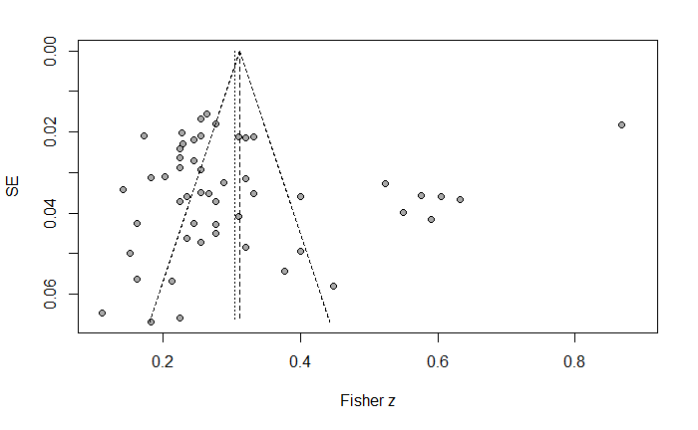
Funnel plot of the association between parental technoference and child problematic media use.

Funnel plots show that effect sizes were roughly evenly distributed on either side of the total effect (Figure 2). The Egger test showed no intercept values in the outcome categories were statistically significant. Specifically, the intercept was –0.78 (*P*=.71). Collectively, these analyses suggest that the findings of this meta-analysis are robust and unlikely to be substantially influenced by publication bias.

### Overall Relation Between Parental Technoference and Child Problematic Media Use

The homogeneity test revealed significant heterogeneity among the effect sizes (*Q*_52_=1558.22; *P*<.001; *I*^2^=96.7%). These findings indicate that there was substantial variability in the effect sizes, suggesting the presence of real differences. Consequently, a random effects model was used to examine both the overall effect and the moderating effect.

**Figure 3:**
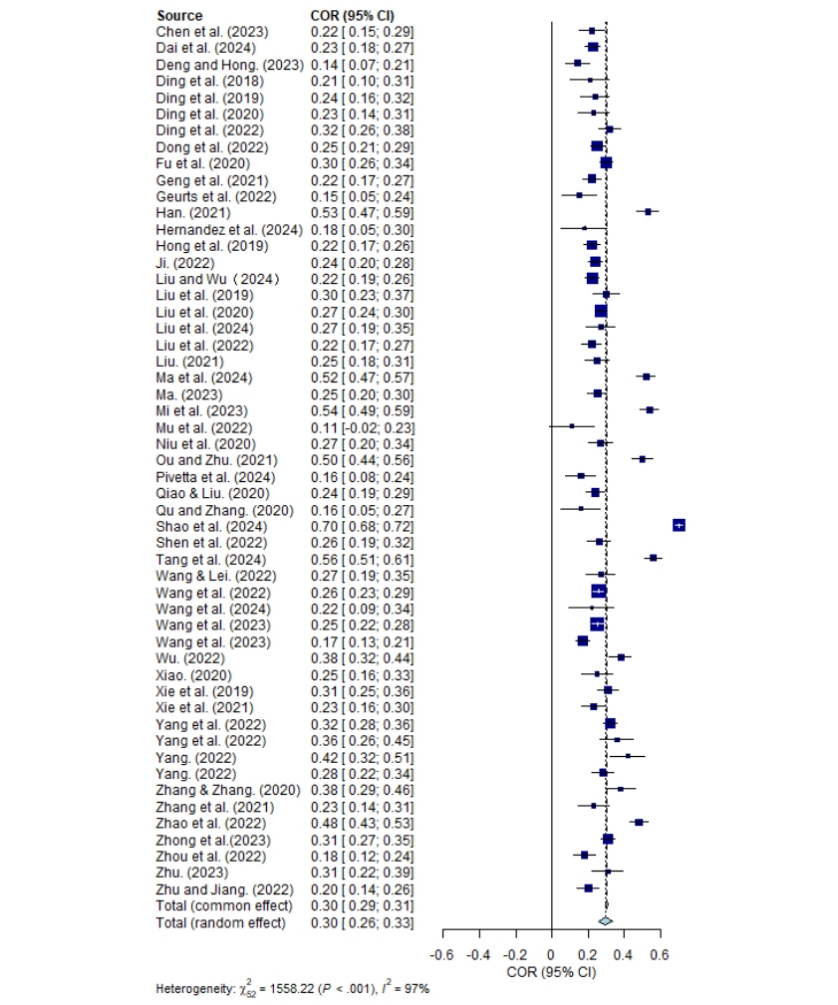
Forest plot for correlation between parental technoference and child problematic media use.

The analysis showed a significant positive correlation between parental technoference and child problematic media use (*r*=0.296; 95% CI 0.259-0.331), as shown in Figure 3 [2,16-18,27-44,50-80]. According to Lipsey and Wilson [83], correlation coefficients can be categorized as low (|*r*|≤0.1), medium (0.1<*|r*|<0.4), or high (|*r*|≥0.4). In this study, the mean effect sizes of the correlation between parental technoference and child problematic media use were considered medium.

### Analysis of Moderator Variables

The results of the meta-regression analysis indicated that the moderating effects of gender (*B*=0.123; SE 0.341; 95% CI 0.546 to 0.792; *z*=0.360; *P*=.72) and age (*B*=0.004; SE 0.010; 95% CI 0.015 to 0.022; *z*=0.368; *P*=.71) was found not significant.

Table 2 presents the detailed analysis results for the categorized moderating variables. The analysis revealed no significant moderating effects for publication status (*P*=.39) or the type of problematic media use (*P*=.24). However, the parental technoference group emerged as a significant moderator (*P*<.001), indicating that the effect size of technoference on children’s problematic media use was larger when both parents engaged in technoference compared to when only one parent exhibited such behavior. Furthermore, the study design demonstrated a significant moderating effect (*P*=.008). Specifically, cross-sectional studies yielded larger effect sizes for the relationship between parental technoference and child problematic media use compared to longitudinal studies.

**Table 2:** Categorical moderator analysis of parental phubbing and internalizing problems.

Moderator variables		Studies, n	*r*	95% CI	*Q* test (*df*)		*P* value	
**Parental technoference group**						18.08 (2)		<.001
	Both parents	44	0.313	0.272-0.354				
	Father	8	0.211	0.182-0.239				
	Mother	8	0.208	0.171-0.246				
**Publication status**						0.73 (1)		.39
	Journal paper	44	0.290	0.248-0.330				
	Thesis	9	0.325	0.254-0.392				
**Study design**						6.84 (1)		.008
	Cross-sectional	46	0.306	0.265-0.345				
	Longitudinal	7	0.223	0.174-0.270				
**Type of PMU^a^**						1.38 (1)		.24
	General problematic media use	42	0.305	0.261-0.348				
	Specific problematic media use	12	0.211	0.202-0.316				

^a^PMU: problematic mobile use.

### Sensitivity Analysis

Furthermore, sensitivity analysis was performed to assess the impact of each study using the leave-one-out method. The sensitivity analysis results indicated that the effect size *r* remained significant, ranging from 0.275 to 0.307, even after excluding any single sample. This suggests that the findings of this study remained relatively stable and reliable.

## Discussion

### Principal Findings

Despite the growing body of empirical research exploring the relationship between parental technoference and child problematic media use, inconsistencies in research conclusions persist. No meta-analysis had been conducted to systematically synthesize these findings—until now. This study used a meta-analytical approach to quantitatively aggregate existing research, examining the relationship between parental technoference and child problematic media use, as well as identifying moderating factors within this relationship. The results indicated a positive association between parental technoference and child problematic media use. Furthermore, moderator analyses showed that the parental technoference group and study design significantly influenced this relationship.

#### Parental Technoference and Child Problematic Media Use

The present meta-analysis provides compelling evidence for a significant positive correlation between parental technoference and child problematic media use. This finding aligns with the interactional theory of childhood problematic media use [19], which posits that children’s media use patterns are influenced by their interactions with their environment, including parental behaviors. Parental technoference, characterized by reduced parent-child interaction and unclear media usage rules [11,20-22], may lead children to engage in excessive media use as a means of compensating for the lack of parental attention and communication.

Moreover, the accept-rejection theory [25] offers another perspective on the relationship between parental technoference and child problematic media use. When parents are preoccupied with technology, children may perceive this as a form of rejection or neglect. Consequently, children may turn to excessive media use as an alternative source of comfort and stimulation to cope with the perceived lack of emotional connection with their parents.

Furthermore, social learning theory [26] suggests that children learn by observing and imitating the behaviors of their parents. When parents engage in excessive media use or prioritize technology over other activities, they inadvertently model problematic media use behaviors. As a result, children may adopt similar problematic media use patterns through observational learning processes.

This meta-analysis, in conjunction with the theoretical perspectives discussed, provides a more comprehensive understanding of the potential mechanisms underlying the relationship between parental technoference and child problematic media use. However, it is important to acknowledge that the strength of this association may vary across studies due to methodological differences, such as sample characteristics, measurement tools, and cultural contexts [16,17]. Future research should aim to further elucidate these factors and their role in modulating the relationship between parental technoference and child problematic media use.

In conclusion, the meta-analysis substantiates the theoretical assertions that parental technoference is a significant contributor to child problematic media use. This relationship can be understood through the lenses of the interactional theory of childhood problematic media use, accept-rejection theory, and social learning theory, which collectively suggest that parental technoference may disrupt parent-child interactions, contribute to children's perception of rejection, and model problematic media use behaviors.

#### Moderator Analysis

The significant moderating effect of the parental technoference group on the relationship between parental technoference and child problematic media use suggests a compounded risk when both parents are involved in technoference. This finding aligns with Zhang et al [84], who reported a stronger association between parental technoference and children’s internalizing problems when both parents, rather than one, engaged in technoference. Our finding extends this understanding to problematic media use, indicating that the combined technoference of both parents may create a more pervasive environment of digital distraction, which could amplify negative outcomes in children’s media behaviors. This could be due to a cumulative lack of parental attention and modeling of poor media habits, indicating the necessity for interventions that address family media use as a whole. Future research should explore the mechanisms through which the combined technoference of both parents exerts a greater influence on child outcomes, potentially informing targeted family-based strategies to mitigate problematic media use in children.

The significant moderating effect of study design, with cross-sectional studies yielding larger effect sizes compared to longitudinal studies, suggests that the impact of parental technoference on child problematic media use may diminish over time. This finding highlights the potential for adaptation or the development of coping mechanisms in children exposed to parental technoference. It also emphasizes the importance of longitudinal research to capture the dynamic nature of this relationship and to inform long-term intervention strategies.

The study did not find significant moderating effects for child gender, age, publication status, or type of problematic media use. This indicates that parental technoference exerts a consistent impact on child problematic media use across different genders, ages, and types of media use issues. Such consistency underscores the pervasive influence of parental technoference, suggesting its stable impact on children’s media behaviors irrespective of individual or contextual differences.

#### Limitations and Future Directions

This meta-analysis provides valuable insights into the relationship between parental technoference and child problematic media use. However, several limitations warrant consideration. The predominance of Chinese participants in the sample limits the generalizability of findings to other cultural contexts, particularly those with differing parenting dynamics and societal values. Future research should encompass a broader cultural spectrum to assess the global applicability of these results. Moreover, the reliance on cross-sectional studies in the analyzed literature presents challenges in establishing causality and may introduce recall biases. To address these limitations, future studies should use experimental methods to isolate the direct impact of parental technoference on child outcomes. Additionally, longitudinal designs spanning extended periods would enable the examination of developmental trajectories and the dynamic interplay between parental technoference and child problematic media use over time. Implementing these methodological approaches in future research will enhance our understanding of the causal mechanisms and long-term implications of parental technoference, ultimately informing more effective interventions to promote healthy family media use practices.

#### Conclusions

This meta-analysis provides robust evidence for a significant positive association between parental technoference and child problematic media use. The findings reveal that this relationship is particularly pronounced when both parents engage in technoference and are stronger in cross-sectional studies compared to longitudinal ones. These results underscore the pervasive influence of parental digital distraction on children’s media habits across various demographics and types of media use. The study highlights the critical need for family-based interventions targeting parental technology use and emphasizes the importance of longitudinal research to understand the temporal dynamics of this relationship better. By addressing parental technoference, we may significantly impact children’s problematic media use, potentially improving developmental outcomes and overall well-being in the digital age. Future research should focus on developing targeted interventions and exploring the underlying mechanisms of this relationship across diverse cultural contexts.

## Appendix

Multimedia Appendix 1 PRISMA-IPD (Preferred Reporting Items for Systematic Review and Meta-Analyses of Individual Participant Data) checklist.

Multimedia Appendix 2 Search strategy for all databases.

Multimedia Appendix 3 Quality assessment rating for each study included in the meta-analysis.

## Abbreviations

ICC: intraclass correlation coefficient
PRISMA: Preferred Reporting Items for Systematic Reviews and Meta-Analyses

## Data Availability

The datasets used or analyzed during the current study are available from the corresponding author upon reasonable request.
