# Supplementary material for: Parental Technoference and Child Problematic Media Use: Meta-Analysis
Source: J Med Internet Res. 2025 Jan 22;27:e57636. doi: 10.2196/57636 (PMC11799820; doi:10.2196/57636)
Supplement: Multimedia Appendix 2 [file jmir_v27i1e57636_app2.docx]

**Search Strategy for All Databases**

**Web of Science**

**(parent* OR parental* OR caregiver* OR guardian* OR dad* OR father* OR mom* OR mother* OR family)** (Topic) and **(child* OR infant* OR baby OR babies OR toddler* OR preschool* OR kid* OR youth* OR teen* OR adolescent* OR young*)** (Topic) and **(technoference* OR phubbing* OR "technology interference*" OR "distraction with phone*" OR "digital distraction*" OR "smartphone distraction*" OR "device distraction*" OR "technology interruption*" OR "digital interruption*" OR "smartphone interruption*" OR "device interruption*" OR "parental media use*" OR "parental smartphone use*" OR "parental device use*")** (Topic) and **Preprint Citation Index** (Exclude – Database)

<https://webofscience.clarivate.cn/wos/alldb/summary/e9626be8-0366-4977-8680-54def7e2283a-01015952d1/relevance/1>

**EBSCO**

**(parent* OR parental* OR caregiver* OR guardian* OR dad* OR father* OR mom* OR mother* OR family)** (Topic) and **(child* OR infant* OR baby OR babies OR toddler* OR preschool* OR kid* OR youth* OR teen* OR adolescent* OR young*)** (Topic) and **(technoference* OR phubbing* OR "technology interference*" OR "distraction with phone*" OR "digital distraction*" OR "smartphone distraction*" OR "device distraction*" OR "technology interruption*" OR "digital interruption*" OR "smartphone interruption*" OR "device interruption*" OR "parental media use*" OR "parental smartphone use*" OR "parental device use*")** (Topic)

https://search.ebscohost.com/login.aspx?direct=true&db=aph&db=pdh&db=pzh&db=pxh&db=psyh&db=aci&db=awr&db=asu&db=qbh&db=buh&db=e5h&db=nlebk&db=e63sww&db=eue&db=enr&db=egs&db=eih&db=eric&db=hev&db=8gh&db=30h&db=hjh&db=hus&db=lls&db=lxh&db=lkh&db=cmedm&db=nfh&db=ddu&db=poh&db=pbh&db=bwh&db=sih&db=s3h&db=trh&bquery=(+(parent*+OR+parental*+OR+caregiver*+OR+guardian*+OR+dad*+OR+father*+OR+mom*+OR+mother*+OR+family)+)+AND+(+(child*+OR+infant*+OR+baby+OR+babies+OR+toddler*+OR+preschool*+OR+kid*+OR+youth*+OR+teen*+OR+adolescent*+OR+young*)+)+AND+(+(technoference*+OR+phubbing*+OR+%26quot%3btechnology+interference*%26quot%3b+OR+%26quot%3bdistraction+with+phone*%26quot%3b+OR+%26quot%3bdigital+distraction*%26quot%3b+OR+%26quot%3bsmartphone+distraction*%26quot%3b+OR+%26quot%3bdevice+distraction*%26quot%3b+OR+%26quot%3btechnology+interruption*%26quot%3b+OR+%26quot%3bdigital+interruption*%26quot%3b+OR+%26quot%3bsmartphone+interruption*%26quot%3b+OR+%26quot%3bdevice+interruption*%26quot%3b+OR+%26quot%3bparental+media+use*%26quot%3b+OR+%26quot%3bparental+smartphone+use*%26quot%3b+OR+%26quot%3bparental+device+use*%26quot%3b)+)&dli0=LA99&dlv0=Chi&dld0=aph&type=1&searchMode=Standard&site=ehost-live

**PubMed and PsycINFO**

("parent*"[All Fields] OR "parental*"[All Fields] OR "caregiver*"[All Fields] OR "guardian*"[All Fields] OR "dad"[All Fields] OR "father*"[All Fields] OR "mom"[All Fields] OR "mother*"[All Fields] OR ("familialities"[All Fields] OR "familiality"[All Fields] OR "familially"[All Fields] OR "familials"[All Fields] OR "familie"[All Fields] OR "family"[MeSH Terms] OR "family"[All Fields] OR "familial"[All Fields] OR "families"[All Fields] OR "family s"[All Fields] OR "familys"[All Fields])) AND ("child*"[All Fields] OR "infant*"[All Fields] OR ("infant, newborn"[MeSH Terms] OR ("infant"[All Fields] AND "newborn"[All Fields]) OR "newborn infant"[All Fields] OR "baby"[All Fields] OR "infant"[MeSH Terms] OR "infant"[All Fields]) OR ("baby s"[All Fields] OR "babys"[All Fields] OR "infant"[MeSH Terms] OR "infant"[All Fields] OR "babies"[All Fields]) OR "toddler*"[All Fields] OR "preschool*"[All Fields] OR "kid"[All Fields] OR "youth*"[All Fields] OR "teen*"[All Fields] OR "adolescent*"[All Fields] OR "young*"[All Fields]) AND ("technoference*"[All Fields] OR "phubbing*"[All Fields] OR "technology interference*"[All Fields] OR (("distract"[All Fields] OR "distractability"[All Fields] OR "distractable"[All Fields] OR "distracted"[All Fields] OR "distracter"[All Fields] OR "distracters"[All Fields] OR "distractibility"[All Fields] OR "distractible"[All Fields] OR "distracting"[All Fields] OR "distraction"[All Fields] OR "distractional"[All Fields] OR "distractions"[All Fields] OR "distractive"[All Fields] OR "distracts"[All Fields]) AND "phone*"[All Fields]) OR "digital distraction*"[All Fields] OR "smartphone distraction*"[All Fields] OR "device distraction*"[All Fields] OR "technology interruption*"[All Fields] OR "digital interruption*"[All Fields] OR (("smartphone"[MeSH Terms] OR "smartphone"[All Fields] OR "smartphones"[All Fields] OR "smartphone s"[All Fields]) AND "interruption*"[All Fields]) OR "device interruption*"[All Fields] OR "parental media use*"[All Fields] OR "parental smartphone use*"[All Fields] OR "parental device use*"[All Fields])

**ProQuest**

[(parent* OR parental* OR caregiver* OR guardian* OR dad* OR father* OR mom* OR mother* OR family) AND (child* OR infant* OR baby OR babies OR toddler* OR preschool* OR kid* OR youth* OR teen* OR adolescent* OR young*) AND (technoference* OR phubbing* OR "technology interference*" OR "distraction with phone*" OR "digital distraction*" OR "smartphone distraction*" OR "device distraction*" OR "technology interruption*" OR "digital interruption*" OR "smartphone interruption*" OR "device interruption*" OR "parental media use*" OR "parental smartphone use*" OR "parental device use*") AND stype.exact("Scholarly Journals" OR "Dissertations & Theses") AND la.exact("English" OR "Chinese")](https://www.proquest.com/myresearch/savedsearches.checkdbssearchlink:rerunsearch/2676965/SavedSearches/$N?site=psychology&t:ac=SavedSearches)

<https://www.proquest.com/search/2676965?accountid=13819>

**CNKI**

| Search Category | Search Terms |
| --- | --- |
| 1. Parental | (父母 OR 亲子 OR 家长 OR 监护人 OR 爸爸 OR 父亲 OR 妈妈 OR 母亲 OR 家庭) |
| 1. Child | (儿童 OR 婴儿 OR 幼儿 OR 学龄前儿童 OR 少年 OR 青少年 OR 青年) |
| 1. Technoference | (科技干扰 OR 低头族 OR 技术干扰 OR 手机干扰 OR 数字干扰 OR 智能手机干扰 OR 设备干扰 OR 科技中断 OR 数字中断 OR 智能手机中断 OR 设备中断 OR 父母媒体使用 OR 父母智能手机使用 OR 父母设备使用) |
| 1. Combind | 1 AND 2 AND 3 |
